# Functional Outcomes and Quality of Life for Patients With Cachexia and Solid Tumour Cancers: Findings of a Systematic Literature Review

**DOI:** 10.1002/jcsm.70319

**Published:** 2026-06-29

**Authors:** Jeffrey Crawford, Marie Fallon, Jarjieh Fang, John D. Groarke, Karen Smoyer, Tateaki Naito, Ira A. Jacobs

**Affiliations:** ^1^ Duke Cancer Institute Duke University Medical Center Durham North Carolina USA; ^2^ Edinburgh Cancer Research Centre, Institute of Genetics and Cancer University of Edinburgh Edinburgh UK; ^3^ Patient‐Centered Outcomes Assessment Pfizer Inc New York New York USA; ^4^ Pfizer Internal Medicine Research Unit Pfizer Inc Cambridge Massachusetts USA; ^5^ Envision Pharma Group Raleigh North Carolina USA; ^6^ Cancer Supportive Care Center, Shizuoka Cancer Center Shizuoka Japan; ^7^ Formerly Pfizer Research and Development Pfizer Inc New York New York USA

**Keywords:** cachexia, cancer, health‐related quality of life, physical function, weight loss

## Abstract

**Background:**

Cachexia, a complex multifactorial syndrome characterized by loss of skeletal muscle mass, is common in cancer and impacts treatment response, quality of life (QoL) and survival. No effective therapy is currently available. This report summarizes findings from the literature on the relationship between cachexia and physical function, activities of daily living and health‐related QoL in patients with solid tumours.

**Methods:**

We conducted a systematic literature review by searching Embase, MEDLINE and Cochrane Library publications from 2018–2023 for relevant studies. After screening and data extraction, a narrative synthesis was performed to identify QoL and functional outcomes in relation to cachexia.

**Results:**

Forty publications representing 37 unique studies and 52 053 patients were identified, with 35 (94.6%) observational studies and two (5.4%) post hoc analyses of randomized trials ranging in sample size from 38 to approximately 17 000 patients. Mean/median patient age ranged from 45 to 79.6 years. Across the 40 publications, 11 different definitions of cachexia or body weight loss were used (with the most common, the Fearon et al. 2011 International Consensus criteria, used in 18 publications [45.0%]), revealing an overall lack of consensus on the most suitable diagnostic criteria for cachexia. Nineteen outcome types were reported, including physical function measures in 31 studies (77.5%), health‐related QoL in 24 studies (60.0%), performance status in 16 studies (40.0%), pain and fatigue in 15 studies (37.5%) each, depression or anxiety in nine studies (22.5%) and activities of daily living in six studies (15.0%). Eleven outcome types were exclusively evaluated in univariate analysis; eight were evaluated in multivariate analysis. Many studies identified statistically significantly worse physical function, activities of daily living or health‐related QoL in patients with cachexia or body weight loss compared with patients without these conditions. Of studies assessing physical function measures, 80.6% (25/31) identified a statistically significant association with cachexia or body weight loss in at least one outcome; for studies assessing health‐related QoL, this was 91.7% (22/24); for performance status, 87.5% (14/16); for pain, 78.6% (11/14); for fatigue, 73.3% (11/15); for depression or anxiety, 55.6% (5/9); and for activities of daily living, 100% (6/6).

**Conclusions:**

This systematic literature review provides insights into functional outcomes and health‐related QoL in predominantly real‐world populations with cancer cachexia and can inform selection of cachexia clinical trial endpoints that reflect clinical benefits to patients. However, the wide range of methods, physical function metrics and patient‐reported outcomes instruments used across studies support a call for standardization.

## 1 Introduction

The global burden of cancer incidence and mortality is increasing. A report from 2022 estimated the cancer burden worldwide being responsible for almost one in six deaths overall (16.8%) and one in four deaths from noncommunicable diseases (22.8%) [1]. About one‐third of cancer deaths are considered attributable to cancer‐associated cachexia [2, 3], a metabolic disorder associated with a progressive loss of body weight [4]. In addition, over half of all patients with cancer have some degree of cachexia at the time of their death [2, 3]. Depending on how cachexia is diagnosed and defined, prevalence estimates vary. A recent systematic literature review (SLR) and meta‐analysis [5] identified a prevalence of 33.0% (95% confidence interval [CI], 32.8, 33.3) based on the International Consensus criteria defining cancer cachexia developed by Fearon et al. in 2011 [6] but reported a broader range of 13.9%–56.5% if using other diagnostic criteria [5].

Cancer‐associated cachexia can arise as cancer progresses, as well as resulting from certain treatment modalities such as chemotherapy [7]. As with cachexia associated with other chronic diseases, such as heart failure, chronic obstructive pulmonary disease and kidney failure, cancer‐associated cachexia is a complex multifactorial syndrome characterized by loss of appetite (anorexia), unintentional body weight loss (WL) and decreased skeletal muscle mass that cannot be fully reversed by conventional nutritional support [6, 8, 9]. In addition to body composition changes, biochemistry changes reflecting systemic inflammation, as measured by C‐reactive protein, serum albumin and neutrophil‐to‐lymphocyte ratio, are also hallmarks of cancer‐associated cachexia [10].

The inability of standard nutritional supplementation to reverse the cachectic process distinguishes cachexia from simple malnutrition and starvation, which can be readily reversed following the provision of adequate nutrition [7]. The consequences of cachexia, particularly in the context of cancer where it is exacerbated by chemotherapy and radiotherapy treatment modalities, can be a substantial burden for patients. For example, cachexia can lead to fatigue, functional impairment, treatment failure, treatment‐related toxicity, poor quality of life (QoL) and reduced survival [9, 11, 12, 13, 14, 15].

The treatment and management of cancer cachexia represents an urgent unmet medical need because no effective therapy is currently available, despite the identification of several molecular mechanisms [16]. In this respect, several investigational clinical trials are underway to investigate potential treatments for cancer cachexia [17, 18]. Such studies frequently use measures of physical function as trial endpoints, which was the subject of a 2023 systematic review by McDonald et al. [19]. This underlines the importance of providing a more detailed description of the relationship between cachexia and physical function.

## 2 Objective

This manuscript reports a summary of an SLR for which the primary objective was to assess the impact of cachexia or unintentional WL on functional outcomes, including physical function and activities of daily living (ADLs), as well as health‐related QoL (HRQoL), in patients with solid tumour cancers, based on observational studies and clinical trials. Definitions for the populations of interest are described in Table 1.

**TABLE 1:** Cancer cachexia and unintentional weight loss populations of interest.

Cancer cachexia‐international consensus definition [6]
Definition as described in Dunne et al. (2019) [20], p.2, for patients with cancer: > 5% weight loss in the previous 6 months Or > 2% weight loss in the previous 6 months *and* one of the following: (1) BMI < 20.0 kg/m^2^ (2) Evidence of muscle depletion (sarcopenia), for example: Appendicular skeletal muscle index determined by dual energy x‐ray absorptiometry (men < 7.26 kg/m^2^; women < 5.45 kg/m^2^ Mid upper‐arm muscle area determined by anthropometry (men < 32 cm^2^, women < 18 cm^2^) Lumbar skeletal muscle index determined by computed tomography imaging (men < 55 cm^2^/m^2^; women < 39 cm^2^/m^2^) Whole body fat‐free muscle mass index without bone determined by bioelectrical impedance (men < 14.6 kg/m^2^; women < 11.4 kg/m^2^) Absolute muscularity below the 5th percentile
Cancer cachexia or unintentional weight loss‐broad definition
Any one of the following, for patients with cancer: Presence of cachexia (any definition or undefined) Any weight loss BMI < 20 kg/m^2^ and one of the following sarcopenia indicators, for patients with cancer Presence of sarcopenia (any definition or undefined) Any skeletal muscle depletion Low muscle density or any reduction in muscle density Low muscle strength^a^ Low muscle quantity or quality^a^ Poor physical performance^a^

Abbreviation: BMI, body mass index.

^a^ Sarcopenia indicators as reported in the Revised European Working Group on Sarcopenia in Older People (EWGSOP2) [21].

## 3 Methods

This SLR was conducted using a standardized methodical, thorough, and transparent approach following guidance presented in the Preferred Reporting Items for Systematic Reviews and Meta‐Analyses (PRISMA) 2020 statement [22]. The SLR processes and methodology follow the PRISMA Protocol (PRISMA‐P) guidelines [23].

This SLR was prospectively registered on the International Prospective Register of Systematic Reviews (PROSPERO), a database of prospectively registered systematic reviews in health and social care, welfare, public health, education, crime, justice and international development. The PROSPERO registration number for this study is PROSPERO 2023 CRD42023485701, available from https://www.crd.york.ac.uk/PROSPERO/view/CRD42023485701.

### 3.1 Eligibility Criteria

The SLR involved a rigorous and objective search and selection process following prespecified inclusion/exclusion criteria to capture the most relevant publications, as outlined in the search strategy reported in Supporting Information Tables S1a–S1c. Included studies were defined according to the Population, Intervention, Comparator, Outcome and Study Type (PICOS) framework using the eligibility criteria outlined in Table 2, including clinical trials, real‐world studies and observational studies. Studies published in English from January 1, 2018, to November 22, 2023, were eligible for inclusion.

**TABLE 2:** Study inclusion criteria.

	Inclusion criteria	Exclusion criteria
Population	Adults and children with solid tumour cancers with any degree of weight loss or cancer (any definition or not defined) BMI < 20.0 kg/m^2^ and an indicator of sarcopenia^a^	Studies with data on haematological malignancies only and no solid tumour outcomes^b^
Intervention/comparator	Any or none (other than those excluded)	Parenteral (IV) or tube fed nutrition^c^ Surgery/resection
Outcomes	Reported as either a change over time or comparison of persons with cachexia/unintentional WL versus without cachexia/unintentional WL: Objective measures of physical function such as exercise capacity, hand grip strength, muscle endurance, walking distance stair climb, actigraphy measures Clinician assessed performance status (ECOG/WHO or Karnofsky PS) Activities of daily living (e.g., Barthel Index) Patient reported outcomes including measures of physical, emotional or social well‐being (e.g., EQ‐5D, SF‐36, FACT, EORTC QLQ instruments or appetite, swallowing etc) Secondary outcome: point at which patients require parenteral or tube‐fed nutrition Aetiology of weight loss (e.g., TRAE, non‐TRAE or unspecified)	Studies not reporting outcomes for groups with cachexia versus without cachexia or cachexia over time Outcomes from patients receiving parenteral (IV) or tube fed nutrition^c^
Study types	Randomized or non‐randomized clinical trials Retrospective or prospective real‐world/observational studies	Preclinical, animal, pilot or phase 1 clinical studies, protocols (without results), case reports/studies Notes, commentaries, letters, editorials, opinions, economic model studies Meta‐analyses, reviews^d^ Conference abstracts
Other	Published in English between 2018 and 2023 (last 5 years)	Publications not in English or published prior to 2018

Abbreviations: BMI, body mass index; ECOG, Eastern Cooperative Oncology Group; EORTC QLQ, European Organisation for Research and Treatment of Cancer quality of life questionnaire; EQ‐5D, EuroQol 5 Dimensions; FACT, Functional Assessment of Cancer Therapy; IV, intravenous; PICOS, Population, intervention, comparator, outcome, study design; PS, performance status; SF‐36, Short Form (36) Health Survey; TRAE, treatment‐related adverse event; WHO, World Health Organization; WL, weight loss.

^a^ To include International Consensus or broad definition (e.g., any WL or cachexia (any or no definition) or BMI < 20.0 kg/m^2^ and sarcopenia for cachexia) as noted in Table 1.

^b^ Studies reporting mixed solid tumours/haematological malignancies included.

^c^ Oral nutrition or supplements included.

^d^ Reviews excluded but reference lists for relevant systematic reviews screened for primary sources.

For the purposes of the SLR, populations with cancer described as having one of the following were considered to have ‘cachexia’: cachexia (undefined or any definition provided by researchers of included publications), any WL or body mass index (BMI) < 20.0 kg/m^2^ accompanied by sarcopenia as defined by one of the following: sarcopenia (any definition or undefined), low/reduced muscle strength, skeletal muscle mass or muscle density. Referring to both ‘cachexia’ and ‘WL’ to describe patient populations of interest reflects the methodologies in the literature sources included; however, it must be noted that these two terms are not synonymous.

### 3.2 Data Sources and Search Strategy

Searches were performed via Ovid on November 16, 2023, in the following databases: Embase; MEDLINE Epub Ahead of Print and In‐Process, In‐Data‐Review & Other Non‐Indexed Citations; Cochrane Library via Evidence Based Medicine (EBM) Reviews (Cochrane Central Register of Controlled Trials [CENTRAL]; Cochrane Database of Systematic Reviews [CDSR]).

Detailed search strategies are provided in Supporting Information Tables S1a–S1c. Results from each database were downloaded to EndNote bibliographic software, with duplications removed in the EndNote database using EndNote algorithms and by hand. To ensure transparency, duplicate citations were retained.

### 3.3 Screening

After removal of duplicates from the combined literature database searches, a single researcher prescreened the hits in the EndNote database to exclude records that were noticeably irrelevant, for example, those relating to animal studies or preclinical trials. Excluded records were checked by a second researcher and retained in an EndNote folder.

After prescreening, a single reviewer conducted level 1 title and abstract screening of the references for eligibility according to the inclusion and exclusion criteria. A second reviewer screened 20% of the level 1 list. Where discrepancies existed, the reference was included in full‐text screening. Full‐text articles were obtained for the citations with titles/abstracts that met the criteria for inclusion.

At the level 2 screen, a single reviewer screened the full texts of publications selected at level 1 against the same inclusion and exclusion criteria. Reasons for exclusion were provided. A second reviewer screened 20% of the publications. Where discrepancies existed, the two reviewers discussed their reasoning until consensus was reached. If required, the senior researcher was consulted to make a final decision. In addition to database screening, a hand search of reference lists from relevant reviews and papers selected for inclusion in the SLR was implemented to identify any publications, which may not have appeared in the database searches. Identification of papers was represented in a flow diagram as per the PRISMA statement [22].

### 3.4 Data Extraction and Critical Appraisal

After selecting publications of interest based on full‐text screening, data extraction tables were developed using Microsoft Excel, which were designed to capture the study and population details in addition to the outcomes described in the eligibility criteria. A single researcher extracted each data point into the data extraction tables, and a second researcher checked every entry for accuracy against the original material.

A single reviewer conducted critical appraisal/risk of bias for all included studies, which was checked by a second reviewer. For observational longitudinal or prospective studies, quality/risk of bias was assessed using the Newcastle‐Ottawa Scale [24], which also was used for any nonrandomized studies. The quality of observational cross‐sectional studies was assessed using the modified Newcastle‐Ottawa Scale [24, 25]. For randomized controlled trials (RCTs), quality was assessed using the Cochrane Risk of Bias 2 tool (RoB 2) [26].

### 3.5 Data Synthesis

A narrative synthesis was performed to identify the QoL and function outcomes and assess differences between cohorts with cachexia or WL versus those without. A narrative synthesis constitutes the best instrument to synthesize the findings of the studies. Due to the heterogeneous nature of the analytical approaches and outcomes assessed, a meta‐analysis was not feasible.

## 4 Results

**FIGURE 1:**
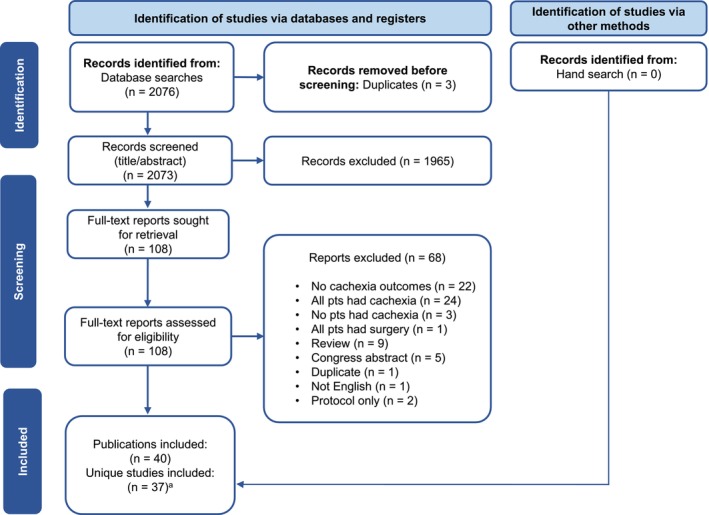
PRISMA diagram showing the screening process. PRISMA, Preferred Reporting Items for Systematic Reviews and Meta‐Analyses; pt, patient. ^a^ Anderson et al. 2020 [27] and Anderson et al. 2021 [28] appear to have some overlap of patients; Cavka et al. 2022 [29] and Cavka et al. 2023 [30], as well as Daly et al. 2020 [31] and Daly et al. 2020 [32], included the same patients but differed in the reporting of outcomes.

In total, 3520 records were identified through the combined database searches; after initial removal of duplicates, 3237 were retained. Prescreening in EndNote removed 1161, resulting in 2076 references uploaded to Covidence screening software, which removed an additional three duplicates. Following level 1 screening, 1965 irrelevant references were removed, leaving 108 full‐text papers, which were assessed for eligibility. After the two‐level screening process was completed, 40 publications representing 37 unique studies and a combined population of 52 053 patients met study inclusion criteria and were retained in the SLR. The most common reasons for publications being excluded at level 2 screening were (1) all patients had cachexia or WL so it was not possible to compare outcomes between two cohorts (*n* = 24) and (2) did not report outcomes for patients with versus without cachexia or WL (*n* = 22). Each step of the screening process, along with reasons for publications being excluded at full‐text screening, is presented in the PRISMA diagram (Figure 1).

### 4.1 Study and Patient Characteristics

Most of the publications were from Asia (*n* = 16) or Europe/the UK (*n* = 16); five studies from the US, two from Latin America and one from Canada were also included. Cross‐sectional analyses were the most common study design (*n* = 18, 45%), followed by prospective cohort studies (*n* = 16, 40%), while four (10%) were retrospective cohort studies and two (5%) were post hoc analyses of RCTs. Sample sizes ranged from 38 to approximately 17 000 patients. Critical appraisal/risk of bias of all 40 publications found that the majority were of moderate or good quality, although many were single‐centre studies and/or had small populations. Critical appraisal of each study is provided in Supporting Information Tables S2a–S2c. Studies exclusively in patients with haematological malignancies were excluded. However, nearly one‐third of studies (*n* = 13, 32.5%) were in patients with mixed solid tumours and haematological malignancies, while nine (22.5%) were in patients with mixed solid tumours. A range of other solid tumour types comprised the remaining studies. Sixty percent of the patients were receiving or had received chemotherapy, with 32.5% receiving chemotherapy combined with radiotherapy. One‐third of studies (*n* = 13, 32.5%) did not mention what type (if any) of cancer therapy patients had received.

The median of reported mean or median age was 61 years and ranged across studies from 45 to 80 years. Except for two studies that included only male patients, the studies were relatively balanced regarding sex. In 12 (30%), more than half of all patients were female, with percentages ranging from 51% to 64%. Across the majority of studies, average patient BMI was within the healthy range, ranging from a mean (standard deviation) BMI of 19.1 (4.5) kg/m^2^ to a median (interquartile range) BMI of 26.9 (24.5–30.0) kg/m^2^.

### 4.2 Cachexia Definitions

**FIGURE 2:**
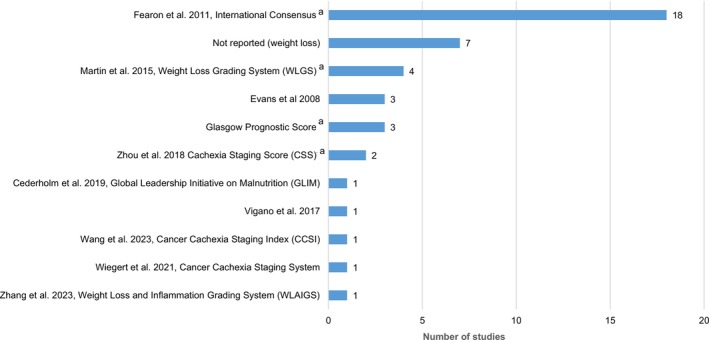
Cachexia definitions used in included publications (*N* = 40). ^a^ Includes modified versions of these scoring systems.

Across the 40 publications, 11 different definitions were used to identify cachexia or WL (Figure 2). The International Consensus criteria from Fearon et al. [6] were used most often, with 18 publications (45.0%) reporting use of this definition of cachexia. Conversely, seven publications (17.5%) did not incorporate a cachexia definition and instead reported physical function, ADL or HRQoL outcomes by % WL, with categories including ≥ 5%, 5% to < 10% or ≥ 10% WL, typically assessed over the preceding 3 or 6 months. Four publications used the Weight Loss Grading System as defined by Martin et al. [33], which accounts for BMI; three used a modified version of the Glasgow Prognostic Score [34, 35, 36, 37], which incorporates C‐reactive protein and albumin but not WL or BMI; and three utilized the comprehensive Evans et al. criteria [38], which included WL, BMI and biomarkers, in addition to hand grip strength (HGS), fatigue and appetite loss. Zhou et al. [39] developed their own multifactorial Cachexia Staging Score that included WL, patient‐reported SARC‐F data on muscle function and sarcopenia, Eastern Cooperative Oncology Group (ECOG) performance status, anorexia and abnormal serum biochemistry; Ueshima et al. [40] also used Zhou's Cachexia Staging Score. There were five other cachexia definitions used in only one study each, three of which the authors developed themselves (Wang et al.: Cancer Cachexia Staging Index [CCSI] [41], Wiegert et al.: Cancer Cachexia Staging System [42] and Zhang et al.: Weight Loss and Inflammation Grading System [WLAIGS] [43]).

### 4.3 Cachexia Outcomes

Nineteen outcome types, reflecting performance status, physical function, ADL, HRQoL, mental health, pain and fatigue, were reported across the 40 included publications. Of these, 11 were evaluated exclusively in univariate analysis (UVA). The eight outcomes for which at least one study used multivariate analysis (MVA) to assess the association with cachexia or WL were (1) HRQoL assessed with European Organisation for Research and Treatment of Cancer (EORTC) QLQ‐C30 or QLQ‐C15‐PAL, *n* = 8/14 studies, significant in seven (87.5% of MVA); (2) physical function assessed by EORTC QLQ‐C30 or QLQ‐C15‐PAL subscore, *n* = 3/14, significant in one study (33.3% of MVA); (3) ADL assessed by various measures, *n* = 2/6, significant in both MVAs; (4) depression, *n* = 2/9, significant in both MVAs; (5) walking distance, *n* = 2/10, not significant in either MVA; (6) HGS, *n* = 2/14, significant in one MVA; (7) HRQoL as assessed by Functional Assessment of Cancer Therapy (FACT) or Functional Assessment of Anorexia/Cachexia Therapy (FAACT) instruments, *n* = 2/6, both of which were significant in MVA; and (8) fatigue, *n* = 1/16 which was not significant in MVA.

**FIGURE 3:**
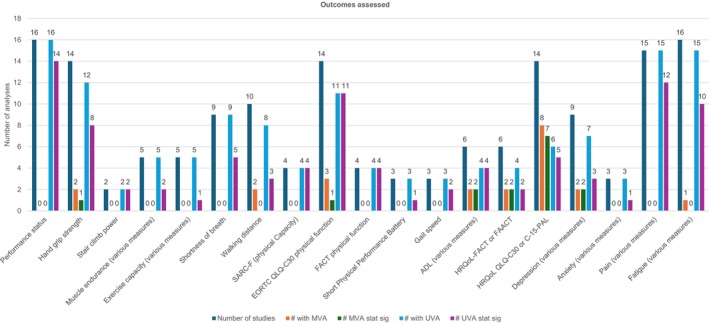
A total of 19 outcome types^a^ assessed for an association with cachexia or weight loss across all included publications (*N* = 40). ADL, activities of daily living; EORTC, European Organization for Research and Treatment of Cancer; FAACT, Functional Assessment of Anorexia/Cachexia Therapy; FACT, Functional Assessment of Cancer Therapy; HRQoL, health‐related quality of life; MVA, multivariate analysis; SARC‐F, Strength, assistance with walking, rising from a chair, climbing stairs and falls; stat sig, statistically significant; UVA, univariate analysis. ^a^ Outcomes assessed in more than 1 study.

For four outcomes (physical function assessed by EORTC QLQ‐C30 subscore; physical function assessed by FACT subscore, either FACT‐General [FACT‐G], FACT‐Prostate [FACT‐P] or FAACT; stair climb power; and SARC‐F, which assesses strength, need for assistance with walking, ability to rise from a chair, ability to climb stairs and fall frequency), 100% of the UVA conducted identified a statistically significant worse outcome for patients with cachexia or WL versus those without these conditions. Details of the numbers and types of outcomes assessed in UVA and in MVA, along with the number of analyses with a statistically significant association between cachexia or WL and the outcome, are shown in Figure 3.

A summary of the main types of outcomes assessed and whether the authors identified a statistically significant association with cachexia or WL for at least one of these outcome types in the study (as studies could have reported more than one measure of an outcome type) is shown in Table 3 and Supporting Information Table S3. Physical function measures were reported most often, with at least one measure assessed in 31 studies (77.5%); of these, a statistically significant association with cachexia or WL was identified in 25 studies (80.6%) assessing physical function. Further details on the specific measures of physical function that were assessed, and their association with cachexia, are shown in Table 4. It should also be noted that many patient‐reported measures of physical function, such as the EORTC QLQ‐C30 physical function scale and FACT‐G, included assessments of ADL as a measure of physical functioning. HRQoL was assessed in 24 studies (60.0%), of which 22 (91.7%) identified statistically worse HRQoL in patients with cachexia or WL versus those without these conditions. Performance status (typically assessed at baseline), measured most often using ECOG performance status, followed by Karnofsky performance status, was reported in 16 studies (40.0%), of which 14 (87.5%) identified a statistically worse performance status in patients with cachexia versus those without cachexia. Pain was assessed in 14 (35%) of the included studies and fatigue in 15 (37.5%), which typically used the corresponding item on the EORTC QLQ‐C30 symptom scale; 78.6% of studies assessing pain and 73.3% of those assessing fatigue identified a statistically significant association with cachexia or WL. Depression or anxiety was infrequently assessed and was only reported in nine studies (22.5%), of which 55.6% identified an association with cachexia or WL. Only six studies (15.0%) reported ADL; all six of these (100%) identified a statistically significant and poorer ability to perform ADL in patients with cachexia or WL versus those without these conditions. Measures assessed included basic self‐care activities and more complex tasks required for independent living (Table 5). Although requirement to switch from oral to enteral or parenteral feeding was an outcome of interest, none of the included studies reported data on this. The majority of assessments of the association between cachexia or WL and one of the outcomes included in this SLR were unadjusted UVA, typically with *p* values reported (Figure 3). Among the studies that assessed statistical significance, most identified worse physical function, ADL or HRQoL in patients with cachexia or WL as compared to those without cachexia or WL. For studies investigating levels of severity, outcomes were generally worse in patients with refractory/more severe cachexia or with WL ≥ 10% (typically assessed over the preceding 3 or 6 months) versus lower percentages of WL.

**TABLE 3:** Summary of outcomes assessed and statistical significance across all included publications (*N* = 40).

Outcome assessed for association with cachexia or WL	Performance status	Physical function	Activities of daily living	HRQoL	Depression and/or anxiety	Pain	Fatigue
Number of publications reporting outcome type, *n* (%) [27, 28, 29, 30, 31, 32, 39, 40, 41, 42, 43, 44, 45, 46, 47, 48, 49, 50, 51, 52, 53, 54, 55, 56, 57, 58, 59, 60, 61, 62, 63, 64, 65, 66, 67, 68, 69, 70, 71, 72]	**16** **(40)**	**31** **(77.5)**	**6** **(15)**	**24** **(60)**	**9** **(22.5)**	**14** **(35)**	**15** **(37.5)**
Number of publications reporting a statistically significant association with cachexia or WL for at least 1 measure of the outcome type,^a^ *n*	**14**	**25**	**6**	**22**	**5**	**11**	**11**

Abbreviations: HRQoL, health‐related quality of life; WL, weight loss.

^a^ Studies may have analysed the same outcome type in different ways or across different patient groups.

**TABLE 4:** Summary of performance status and physical function outcomes assessed and statistical significance across the 40 publications.

Performance status and physical function measures assessed for association with cachexia or WL (32 of 40 publications)	Performance status	HGS	Stair climb power/muscle endurance	Exercise capacity/shortness of breath	Walking distance	SARC‐F	Other
Number of publications reporting outcome type, *n* (%)	16 (40)	13 (32.5)	6 (15)	10 (25)	10 (25)	4 (10)	20 (50)
Number of publications reporting a statistically significant association with cachexia or WL for at least 1 measure of the outcome type, ^a^ *n*	**14**	**9**	**4**	**7**	**5**	**4**	**17**
Summary of performance status and physical function measures assessed across all studies (number of individual studies)^b^; statistically significant associations are shown in **bold**	**ECOG PS (10)** **KPS (5)** — ECOG PS (1) KPS (2)	**HGS (9)** — HGS (5)	**Stair climb power (1)** **1‐rep max strength test (1)** **5 × chair stand test (2)** **One‐leg stance test (1)** **Self‐rated muscle strength scale (1)** — Stair climb power (1) 1‐rep max strength test (1) 5 × chair stand test (1)	**EORTC QLQ‐30 symptom score, dyspnea (6)** **EORTC QLQ‐C15‐PAL, dyspnea (1)** **MDASI, SOB (1)** **Difficulty breathing (1)** — EORTC QLQ‐30 symptom score, dyspnea (2) MDASI, SOB (2) SOB (1)	**MDASI, walking (2)** **Gait speed (2)** **6MWT (1)** **4‐m walk time (1)** **Timed Up and Go test (1)** — Gait speed (1) 6MWT (4) Timed Up and Go test (3) Number of steps (2)	**SARC‐F score (4)**	**EORTC QLQ‐30, physical function (12)** **EORTC QLQ‐C15‐PAL, physical function (1)** **FACT‐G, physical well‐being (2)** **FAACT, physical well‐being (1)** **Self‐rated physical status scale (1)** **SPPB (1)** **IPAQ, activity score (1)** **MDASI, general activity (1)** **Activity time (1)** ‐‐‐‐‐‐‐‐‐‐‐‐ EORTC QLQ‐30, physical function (1) SPPB (2) IPAQ, sedentary score (1) Falls (1)

Abbreviations: 6MWT, 6‐min walk test; ECOG PS, Eastern Cooperative Oncology Group performance status; EORTC, European Organisation for Research and Treatment of Cancer; FAACT, Functional Assessment of Anorexia/Cachexia Therapy; FACT‐G, Functional Assessment of Cancer Therapy‐General; HGS, hand grip strength; IPAQ, International Physical Activity Questionnaire; KPS, Karnofsky performance status; MDASI, MD Anderson Symptom Inventory; rep max, repetition maximum; SOB, shortness of breath; SARC‐F, strength, assistance with walking, rising from a chair, climbing stairs and falls; SPPB, Short Physical Performance Battery; WL, weight loss.

^a^ Studies may have analysed the same outcome type in different ways or across different patient groups.

^b^ Studies may have assessed multiple measures within a category.

**TABLE 5:** Summary of activities of daily living outcomes assessed and statistical significance across the 40 publications.

ADL measures assessed for association with cachexia or WL (6 of 40 publications)	Survey overall score	Individual scores	FIM‐motor	FIM‐cognitive
Number of publications reporting outcome type, *n* (%)	4 (10)	2 (5)	1 (2.5)	1 (2.5)
Number of publications reporting a statistically significant association with cachexia or WL for at least 1 measure of the outcome type, ^a^ *n*	**4**	**2**	**1**	**0**
Summary of ADL measures assessed across all studies (number of individual studies)^b^; statistically significant associations are shown in **bold**	**Barthel Index (1)** **Exercise of Self‐Care Agency (1)** **Basic ADL (1)** **Instrumental ADL (2)**	**Barthel Index measures (1)** **Difficulty swallowing (1)**	**Motor FIM (1)** — Motor FIM (1)	Cognitive FIM (2)

Abbreviations: ADL, activities of daily living; FIM, functional independence measure; WL, weight loss.

^a^ Studies may have analysed the same outcome type in different ways or across different patient groups.

^b^ Studies may have assessed multiple measures within a category.

## 5 Discussion

This SLR of 40 publications representing 37 unique studies from diverse countries provides valuable evidence on the relationship between cachexia or unintentional WL and functional outcomes, including physical function and ADL, as well as HRQoL in patients over a wide range of solid tumour cancers, in addition to some mixed populations that included haematological malignancies. A strength of this SLR is the use of a robust and systematic approach including comprehensive searches across the major medical literature databases, global representation and a study protocol documenting the predefined selection criteria, screening process and plan for data extraction and quality assessment/risk of bias reporting.

There was substantial heterogeneity in study design, patient populations, treatments received and study outcomes, with 11 different cachexia or WL definitions used and 19 different outcome types assessed. Slightly more than half (21/37, 56.8%) of all studies were conducted at a single centre, and sample sizes ranged from small single‐centre studies to very large analyses comprising approximately 17 000 patients. Although the scope of the SLR was global, 80% of the studies were from Asia and Europe (including the UK), with relatively few studies from the US. This may reflect a greater focus on holistic cancer care in these countries.

There was a lack of global consensus regarding different stages or levels of cachexia severity, and many different definitions of cachexia were employed. Although International Consensus criteria based on Fearon et al. [6] are available and were the most frequently used (*n* = 18, 45%), other published cachexia criteria were employed in multiple studies, revealing a lack of consensus on the most suitable diagnostic criteria for cachexia. The included publications used varied cachexia criteria, reporting on either novel classification systems or suggested modifications to an existing cachexia classification system. A case could be made for revisiting the Cachexia International Consensus Criteria to develop updated criteria for widespread adoption. However, it will be important to ensure that any new set of consensus criteria remains sufficiently simple to allow for effective implementation in screening for and diagnosis of cachexia in routine cancer care.

Substantial heterogeneity was identified in the types of outcomes assessed across the 40 publications. Clinician assessments of physical function in terms of performance status, including Karnofsky and ECOG performance scores, as well as objective measures such as 6‐min walk distance, stair climb power, chair stand time, HGS and the Short Physical Performance Battery were used. Physical function was also reported by the physical function subscores of patient‐reported outcomes (PROs) such as the EORTC QLQ‐C30 and patient reports of shortness of breath/dyspnoea as an individual symptom item within a comprehensive PRO instrument. Many of these also included assessments of ADL as a measure of physical functioning. Despite the differences in cachexia definition across the studies meeting eligibility criteria for this SLR, a consistent trend towards impaired physical function and worse HRQoL with increasingly severe cachexia or increased WL was observed, regardless of the definition used.

Information from the predominantly observational studies included in this SLR that provide insights into the physical function and HRQoL of patients with cancer cachexia or unintentional WL in the real‐world setting can also inform selection of clinical trial endpoints for cachexia treatments that reflect benefits to patients. These benefits include symptom reduction and maintenance of physical function, which are important in regulatory review, as well as increased quality‐adjusted life years, which are essential in the reimbursement decision‐making of many national and regional health systems. Changes in physical function and HRQoL, even if inconvenient to collect in trials, are meaningful to patients and clinically relevant.

A 2023 SLR by McDonald et al. described the impact of interventions for cancer cachexia on physical function endpoints in clinical trials. The current SLR, which focuses on the relationship between cachexia per se and physical function measures and is largely based on observational studies, provides a complementary set of results characterizing cachexia in real‐world populations with cancer. McDonald et al. reported that of the types of physical function outcomes included in 71 clinical trials of cancer cachexia interventions, HGS was the most often used (*n* = 33 trials, 46.5%) but was only statistically significant in 12 trials (36%) [19]. In our SLR, for which 94.6% of studies were observational, HGS was also the most frequently reported objective measure of physical function identified (*n* = 13, 32.5% of publications); however, unlike in the clinical trials, HGS was statistically significant in 69.2% of the cachexia or WL associations tested in our SLR. It should also be considered how much HGS affects a patient's HRQoL and ability to engage in ADL. Objective measures of physical function such as stair climb power, chair stand time/timed up and go or 6‐minute walk distance (6MWD) may be more representative of a patient's ability to go about their daily lives, navigate stairs, get out of bed, traverse throughout their home and remain living in the community. However, for these outcome measures, the magnitude of effects that reflect clinical benefit in cachexia clinical trials needs to be better understood.

The most frequently used physical function outcome in our SLR was clinician assessed performance status, as reported in 16 publications (40%) and evaluated entirely by UVA, for which 87.5% of assessments were statistically significantly different between cachexia/WL versus no cachexia/no WL groups. In the McDonald et al. (2023) SLR of 71 cancer cachexia clinical trials, performance status also was a common endpoint, being reported in a total of 25 trials (35.2%). The authors noted that seven of nine trials (78%) assessing ECOG performance status and five of 16 (32%) assessing Karnofsky performance status identified statistically significant changes with receipt of the studied cachexia treatment intervention [19].

A large number of the cachexia/WL assessments of physical function identified in our SLR utilized the patient‐reported physical function subscales of comprehensive PRO instruments, primarily EORTC QLQ‐C30/QLQ‐C15‐PAL (*n* = 14, 35%) and FACT/FAACT (*n* = 3 for each; 7.5%). McDonald et al. noted similar findings, with 25 trials (35.2%) in their SLR reporting the physical function subscale of the EORTC QLQ‐C30, for which 11 (44%) of these were statistically significant [19].

In our SLR, only 15% (*n* = 6) of the cachexia or WL assessments, across five studies, investigated ADL outcomes despite the importance of these activities to a patient's function and ability to live independently. Two studies (Dunne et al. 2019 [54]; Regueme et al. 2021 [63]) used the multidimensional Lawton‐Brody Instrumental ADL, which includes the ability to manage their finances, housekeeping, food preparation, medications and transportation [73]. Although both ECOG performance status and Karnofsky performance status purport to capture some measures of ADL, these are clinician‐assessed rather than patient‐reported, and while they are proven prognostic indicators, they may not accurately reflect a patient's functional independence. In addition, different patient‐reported measures of physical function may reflect distinct aspects of day‐to‐day living and may therefore not necessarily be able to provide universally applicable measures of impact on ADL [19].

Objective measures of physical function, clinician‐rated performance status and patient‐reported physical function measures, while related concepts show substantial variability in how robustly they correlate with cachexia. However, as each of these categories encompasses different individual measures of physical function, comparisons are challenging. Many such measures, including patient‐reported parameters, have only been examined in a limited number of cachexia studies, making it difficult to draw reliable conclusions. There are suggestions that measures such as the 6‐minute walk test, ECOG performance status or Karnofsky performance status may be more sensitive to changes in physical function or have a lower variability than the frequently used HGS test. However, few studies have systematically assessed different approaches to evaluating physical function in the same populations. Thus, further investigation is needed to determine the relationships between measures based on objective physical function, patient‐reported function and clinician‐assessed performance and their relative sensitivity to the severity of cachexia [19]. An optimal approach for diagnosing and monitoring cachexia may involve the complementary use of several different types of physical function measures alongside each other. Determining the best proxy measures for physical function and functional independence in patients with cancer cachexia should be guided by a consensus on which assessments are the most clinically meaningful while also taking the patient perspective into account. A series of studies by Fried et al. (2002–2011) showed that, for a high proportion of patients, the maintenance of ADL was of paramount importance, and for many, the loss of independence and the prospect of severe functional impairment entailed more dread than death [74, 75, 76]. In addition, the debilitating nature of cachexia has been shown to prolong hospital stays in patients with cancer compared with those without cachexia, resulting in a greater healthcare resource burden [77, 78].

HRQoL outcomes were reported in 24 (60%) of the publications included in the SLR, most of which utilized cancer‐specific instruments. The most common PRO instrument was the EORTC QLQ‐C30 (*n* = 13 assessments across 12 studies). Six studies used a FACT instrument (Alvaro Sanz et al. 2021 [45]; Cavka et al. 2022 [29]; Cavka et al. 2023 [30]), including the FAACT (Arrieta et al. 2018 [49]; Roeland et al. 2021 [64]; Zhou et al. 2018 [39]). Two studies (Anderson et al. 2021 [28]; Wang et al. 2023 [41]) measured HRQoL with the cancer‐specific MD Anderson Symptom Inventory. Only one study (Hadzibegovic et al. 2023 [56]) used the EuroQol 5 Dimensions (EQ‐5D).

The varied use of PRO instruments to represent HRQoL among patients with cancer cachexia identified in our SLR of primarily observational studies reflects those of a SLR by Hjermstad et al. (2024) that identified 17 different PROs used across 50 cancer cachexia clinical trials [79]. The authors reported that EORTC QLQ‐C30 was used most often (60% of trials), while 34% used a version of the Functional Assessment of Chronic Illness Therapy (FACIT) or the FACT [79]. Interestingly, in the SLR by Hjermstad et al. (2024), as well as in our SLR, the cachexia‐specific FAACT was used infrequently (*n* = 3, 7.5% of publications).

Overall, we noted a lack of rigorous analytic methods in many studies included in our SLR, as well as a diverse array of physical function metrics and PRO instruments used, which support a call for standardized use of these metrics and PROs in addition to more robust study design and reporting. Although in this SLR, only two of the studies, both post hoc analyses, were based on clinical trials (Regueme et al. 2021 [63]; Stene et al. 2019 [67]) and SLRs investigating PRO use in clinical trials for oncology [80] and for cancer cachexia interventions [79] have noted similar findings. There is a need for improved quality in the design and conduct of physical function metrics and PRO endpoints in oncology clinical trials [80], including prespecified hypotheses for utilizing measures of physical function, ADL or HRQoL in trials and clarity on the minimal clinically important difference for these endpoints. The selection of specific metrics must be informed by which of these measures most accurately reflect the key trial outcomes assessed, which in turn should reflect the aim and mechanism of the intervention studied [19].

Patients with advanced cancer have poorer performance status and HRQoL than the noncancer population, both as a result of the cancer itself as well as cancer treatment [81, 82, 83, 84, 85]. As such, supportive care therapies like granulocyte colony stimulating factor and erythropoietin stimulating agents are part of established treatment guidelines [86, 87]. In a similar manner, the effective management of cancer‐associated cachexia could be added to the battery of options for cancer care, particularly as clinical trials are investigating treatments for cachexia. Addressing cachexia has the potential to improve how patients with cancer function as well as how they feel, in addition to the potential to extend life.

There were some limitations to this SLR. One such limitation was that we did not include publications in languages other than English. In addition, our search was limited to publications between 2018–2023 and therefore cannot reflect the full extent of physical function and QoL data collected over time as part of research into cachexia. Another limitation was the substantial heterogeneity across the included studies, in particular, study design, country and region (which influence healthcare practices and treatment patterns), patient characteristics (e.g., age range, sex, cancer type and stage, time since diagnosis and treatments received), cachexia definitions used and outcomes reported. Therefore, the studies were synthesized qualitatively, and meta‐analysis was not considered. In addition, since the majority of analyses were unadjusted, it is possible that the observed associations could be spurious or weaker than noted once accounting for potential confounders. However, given the large number of studies reporting worse physical function, ADL or HRQoL among patients with cachexia or WL, as well as the plausibility of these findings, it is expected that the association is valid. Additional research utilizing multivariate models, or other means of controlling for confounders, is needed to quantify the associations between cachexia or WL and worse physical function, ADL or HRQoL and confirm that the findings are maintained when assessed with more rigorous methods.

## 6 Conclusions

This SLR assessed physical function, ADL and HRQoL outcomes associated with cachexia or WL in a combined total of 52 053 patients represented in 37 studies and published in 40 peer‐reviewed journal manuscripts. A large number of cachexia or WL definitions were used and numerous physical function, ADL and HRQoL outcomes were assessed, illustrating the range of impacts of cachexia, in terms of how patients with cancer function and feel. Patients with cachexia or WL, regardless of how this condition was measured, were more likely to have worse HRQoL and physical function outcomes than patients without cachexia. The results of this SLR can be used to inform selection of outcome measures in future clinical trials of cancer cachexia. However, more rigorous investigation of these outcomes is warranted, including larger patient populations, gathered from multiple rather than single centres and use of appropriate statistical control of confounding variables. Novel cachexia treatments represent a notable unmet medical need as part of supportive care for patients with cancer and warrant additional research.

## Ethics Statement

The authors comply with the ethical guidelines for authorship and publishing in the Journal of Cachexia, Sarcopenia and Muscle. The manuscript does not contain clinical studies or patient data.

## Conflicts of Interest

Jeffrey Crawford has served as a scientific advisor for Actimed, GeneScience, Pfizer Inc. and Tensegrity Pharma Inc. and as a member of the data safety monitoring board for BioAtla and G1 Therapeutics; and has received research funding from AstraZeneca, Helsinn, Pfizer Inc. and the National Cancer Institute National Clinical Trials Network.

Marie Fallon has served as an advisory board member for Pfizer Inc. Phase 2 and Phase 3 ponsegromab studies and is on the advisory board of Ananda MRX.

Jarjieh Fang and John D. Groarke are employees of Pfizer Inc.

Ira A. Jacobs is a former employee of Pfizer Inc.

Karen Smoyer is an employee and shareholder of Envision Pharma Group.

Tateaki Naito received lecture honoraria from ONO Pharmaceutical Co. Ltd.; research funding from Otsuka Pharmaceutical Co. Ltd. and Kracie Ltd.; and advisory fees from Pfizer Inc., Tensegrity Pharma Inc. and AstraZeneca K.K.

## Supporting information


**Table S1a:** Embase search strategy.
**Table S1b:** MEDLINE search strategy.
**Table S1c:** Cochrane Library search strategies.
**Tables S2a S2c:** jcsm70319‐sup‐0001‐Supporting_Information.docx.
**Table S2a:** Newcastle‐Ottawa Scale for cohort studies (n = 20).
**Table S2b:** Newcastle‐Ottawa Scale for cross‐sectional studies (n = 18).
**Table S2c:** Cochrane Risk of Bias 2 for randomized clinical trials (n = 2). Abbreviations: CI, confidence interval; EMR, electronic medical records; max, maximum; NR, not reported; pt, point; RCT, randomized controlled trial.
**Supporting Information Table:** **S3.** Summary of outcomes assessed and statistical significance across all included publications (N = 40).
