# Supplementary material for: Functional Outcomes and Quality of Life for Patients With Cachexia and Solid Tumour Cancers: Findings of a Systematic Literature Review
Source: J Cachexia Sarcopenia Muscle. 2026 Jun 29;17(4):e70319. doi: 10.1002/jcsm.70319 (PMC13314541; doi:10.1002/jcsm.70319)
Supplement: Supplementary file 1 — Table S1a: Embase search strategy. Table S1b: MEDLINE search strategy. Table S1c: Cochrane Library search strategies. Tables S2a S2c: jcsm70319‐sup‐0001‐Supporting_Information.docx. Table S2a: Newcastle‐Ottawa Scale for cohort studies (n = 20). Table S2b: Newcastle‐Ottawa Scale for cross‐sectional studies (n = 18). Table S2c: Cochrane Risk of Bias 2 for randomized clinical trials (n = 2). Abbreviations: CI, confidence interval; EMR, electronic medical records; max, maximum; NR, not reported; pt, point; RCT, randomized controlled trial. Supporting Information Table: S3. Summary of outcomes assessed and statistical significance across all included publications (N = 40). [file JCSM-17-e70319-s001.docx]

**SUPPORTING INFORMATION**

**Functional Outcomes and Quality of Life for Patients With Cachexia and Solid Tumour Cancers: Findings of a Systematic Literature Review**

*Journal of Cachexia, Sarcopenia and Muscle*

Jeffrey Crawford, Marie Fallon, Jarjieh Fang, John D. Groarke, Karen Smoyer, Tateaki Naito, Ira A. Jacobs.

**Corresponding author:**

Ira A. Jacobs

Email: iraallenjacobs@aol.com

Telephone: + 1 646-915-5019

**Supporting Information Tables S1a-S1c**

**Supporting Information Table S1a.** Embase search strategy.

Embase <1974 to 2023 November 22>. Run November 23, 2023

| **Step** | **Search String** |
| --- | --- |
| 1 | exp cachexia/ |
| 2 | (cachexia or cachectic).mp. |
| 3 | (emaciation or wasting or weight loss or weight reduction).ti,ab. |
| 4 | 1 or 2 or 3 |
| 5 | (neoplasm$ or glioblastoma$ or cancer* or tumour$ or tumor$ or malignan$ or carcinoma$ or adenocarcinoma$ or oncolog$ or gliom$).ti,ab. |
| 6 | 4 and 5 |
| 7 | exp Activities of Daily Living/ |
| 8 | exp daily life activity/ or exp ADL disability/ |
| 9 | functional status/ or functional assessment/ |
| 10 | Barthel index/ |
| 11 | physical performance/ or physical function.mp. |
| 12 | physical mobility/ or mobility.mp. |
| 13 | "activities of daily living".mp. or daily life activity/ |
| 14 | functional independence measure/ |
| 15 | sickness impact profile/ |
| 16 | (ADL* or BADL* or IADL* or EADL*).ti,ab,kw. |
| 17 | (function* adj3 (capacity or independen* or status or disabilit* or assess* or abilit*)).ti,ab,kw. |
| 18 | patient-specific functional scale.ti,ab,kw. |
| 19 | ((daily life or daily living or instrument* or leisure or extended) adj3 activit*).ti,ab,kw. |
| 20 | exercise test/ or (6MWT or 6MWD or 6 min* walk* test or 6 min* walk* distance or six min* walk* test or six min* walk* distance or walk* test*).ti,ab,kw. |
| 21 | (Gait speed or walk speed or "timed get up and go" or chair ris* or hand strength or handgrip strength or grip strength).ti,ab,kw. |
| 22 | ((exercise* or physical) and (capacit* or performance* or endurance* or exertion*)).ti,ab,kw. |
| 23 | (sf36 or sf 36 or short form 36 or shortform 36 or shortform36).ti,ab. |
| 24 | (euroqol* or eq5d* or eq 5d*).ti,ab. |
| 25 | (sf12 or sf 12 or short form 12 or shortform 12 or shortform12).ti,ab. |
| 26 | (sf20 or sf 20 or short form 20 or shortform 20 or shortform20).ti,ab. |
| 27 | (sf8 or sf 8 or short form 8 or shortform 8 or shortform8).ti,ab. |
| 28 | exp "Quality of Life"/ |
| 29 | (quality of life or QoL or QL or HRQoL or HRQL or life quality or health status or health state or health-related or health related or wellbeing or well-being or humanistic).ti,ab,kw. |
| 30 | exp patient-reported outcome/ |
| 31 | ((patient* or proxy or proxies or caregiver*)c adj3 (report* or relate* or perspective* or experience* or satisfaction or burden)).ti,ab,kw. |
| 32 | or/7-31 |
| 33 | 6 and 32 |
| 34 | (infan* or child* or adolescen* or pediatr* or paediatr* or juvenile or pregnan*).ti,ab,kw. |
| 35 | (exp animal/ not (exp human/ and exp animal/)) or exp preclinical study/ |
| 36 | (editorial or comment* or letter or note or case study or case studies or case report).pt. or (editorial/ or letter/ or case study/ or case report/ or note/) or (case report* or case stud*).ti,ab. |
| 37 | conference abstract.pt. |
| 38 | or/34-37 |
| 39 | 33 not 38 |
| 40 | limit 39 to english language |
| 41 | limit 40 to yr="2018 -Current" |

**Supporting Information Table S1b.** MEDLINE search strategy.

Ovid MEDLINE and Epub Ahead of Print, In-Process, In-Data-Review & Other Non-Indexed Citations, Daily and Versions <1946 to November 22, 2023>. Run November 23, 2023

| **Step** | **Search String** |
| --- | --- |
| 1 | exp cachexia/ |
| 2 | (cachexia or cachectic).mp. |
| 3 | (emaciation or wasting or weight loss or weight reduction).ti,ab. |
| 4 | 1 or 2 or 3 |
| 5 | (neoplasm$ or glioblastoma$ or cancer* or tumour$ or tumor$ or malignan$ or carcinoma$ or adenocarcinoma$ or oncolog$ or gliom$).ti,ab. |
| 6 | 4 and 5 |
| 7 | exp Activities of Daily Living/ |
| 8 | (daily life activit* or ADL or ADL disabilit*).mp. |
| 9 | functional status/ or functional assessment.mp. |
| 10 | Barthel index/ |
| 11 | physical performance.mp. or Physical Functional Performance/ |
| 12 | (physical mobilit* or mobility).mp. |
| 13 | "activities of daily living".mp. or daily life activity/ |
| 14 | "functional independence measure".mp. |
| 15 | sickness impact profile/ |
| 16 | (ADL* or BADL* or IADL* or EADL*).ti,ab,kw. |
| 17 | (function* adj3 (capacity or independen* or status or disabilit* or assess* or abilit*)).ti,ab,kw. |
| 18 | patient-specific functional scale.ti,ab,kw. |
| 19 | ((daily life or daily living or instrument* or leisure or extended) adj3 activit*).ti,ab,kw. |
| 20 | exercise test/ or (6MWT or 6MWD or 6 min* walk* test or 6 min* walk* distance or six min* walk* test or six min* walk* distance or walk* test*).ti,ab,kw. |
| 21 | (Gait speed or walk speed or "timed get up and go" or chair ris* or hand strength or handgrip strength or grip strength).ti,ab,kw. |
| 22 | ((exercise* or physical) and (capacit* or performance* or endurance* or exertion*)).ti,ab,kw. |
| 23 | (sf36 or sf 36 or short form 36 or shortform 36 or shortform36).ti,ab. |
| 24 | (euroqol* or eq5d* or eq 5d*).ti,ab. |
| 25 | (sf12 or sf 12 or short form 12 or shortform 12 or shortform12).ti,ab. |
| 26 | (sf20 or sf 20 or short form 20 or shortform 20 or shortform20).ti,ab. |
| 27 | (sf8 or sf 8 or short form 8 or shortform 8 or shortform8).ti,ab. |
| 28 | exp "Quality of Life"/ |
| 29 | (quality of life or QoL or QL or HRQoL or HRQL or life quality or health status or health state or health-related or health related or wellbeing or well-being or humanistic).ti,ab,kw. |
| 30 | exp Patient Reported Outcome Measures/ |
| 31 | ((patient* or proxy or proxies or caregiver*) adj3 (report* or relate* or perspective* or experience* or satisfaction or burden)).ti,ab,kw. |
| 32 | or/7-31 |
| 33 | 6 and 32 |
| 34 | (infan* or child* or adolescen* or pediatr* or paediatr* or juvenile or pregnan*).ti,ab,kw. |
| 35 | (exp animal/ not (exp human/ and exp animal/)) or Drug Evaluation, Preclinical/ |
| 36 | (editorial or comment* or letter or note or case study or case studies or case report).pt. or (editorial/ or letter/ or case study/ or case report/ or note/) or (case report* or case stud*).ti,ab. |
| 37 | conference abstract.pt. |
| 38 | or/34-37 |
| 39 | 33 not 38 |
| 40 | limit 39 to english language |
| 41 | limit 40 to yr="2018 -Current" |

**Supporting Information Table S1c.** Cochrane Library search strategies.

EBM Reviews - Cochrane Central Register of Controlled Trials <October 2023>. Run November 23, 2023

| **Step** | **Search String** |
| --- | --- |
| 1 | exp cachexia/ |
| 2 | (cachexia or cachectic).mp. |
| 3 | (emaciation or wasting or weight loss or weight reduction).ti,ab. |
| 4 | 1 or 2 or 3 |
| 5 | (neoplasm$ or glioblastoma$ or cancer* or tumour$ or tumor$ or malignan$ or carcinoma$ or adenocarcinoma$ or oncolog$ or gliom$).ti,ab. |
| 6 | 4 and 5 |
| 7 | exp Activities of Daily Living/ |
| 8 | (daily life activit* or ADL or ADL disabilit*).mp. |
| 9 | functional status/ or functional assessment.mp. |
| 10 | Barthel index/ |
| 11 | physical performance.mp. or Physical Functional Performance/ |
| 12 | (physical mobilit* or mobility).mp. |
| 13 | "activities of daily living".mp. or daily life activity/ |
| 14 | "functional independence measure".mp. |
| 15 | sickness impact profile/ |
| 16 | (ADL* or BADL* or IADL* or EADL*).ti,ab,kw. |
| 17 | (function* adj3 (capacity or independen* or status or disabilit* or assess* or abilit*)).ti,ab,kw. |
| 18 | patient-specific functional scale.ti,ab,kw. |
| 19 | ((daily life or daily living or instrument* or leisure or extended) adj3 activit*).ti,ab,kw. |
| 20 | exercise test/ or (6MWT or 6MWD or 6 min* walk* test or 6 min* walk* distance or six min* walk* test or six min* walk* distance or walk* test*).ti,ab,kw. |
| 21 | (Gait speed or walk speed or "timed get up and go" or chair ris* or hand strength or handgrip strength or grip strength).ti,ab,kw. |
| 22 | ((exercise* or physical) and (capacit* or performance* or endurance* or exertion*)).ti,ab,kw. |
| 23 | (sf36 or sf 36 or short form 36 or shortform 36 or shortform36).ti,ab. |
| 24 | (euroqol* or eq5d* or eq 5d*).ti,ab. |
| 25 | (sf12 or sf 12 or short form 12 or shortform 12 or shortform12).ti,ab. |
| 26 | (sf20 or sf 20 or short form 20 or shortform 20 or shortform20).ti,ab. |
| 27 | (sf8 or sf 8 or short form 8 or shortform 8 or shortform8).ti,ab. |
| 28 | exp "Quality of Life"/ |
| 29 | (quality of life or QoL or QL or HRQoL or HRQL or life quality or health status or health state or health-related or health related or wellbeing or well-being or humanistic).ti,ab,kw. |
| 30 | exp Patient Reported Outcome Measures/ |
| 31 | ((patient* or proxy or proxies or caregiver*) adj3 (report* or relate* or perspective* or experience* or satisfaction or burden)).ti,ab,kw. |
| 32 | or/7-31 |
| 33 | 6 and 32 |
| 34 | (infan* or child* or adolescen* or pediatr* or paediatr* or juvenile or pregnan*).ti,ab,kw. |
| 35 | (exp animal/ not (exp human/ and exp animal/)) or Drug Evaluation, Preclinical/ |
| 36 | (editorial or comment* or letter or note or case study or case studies or case report).pt. or (editorial/ or letter/ or case study/ or case report/ or note/) or (case report* or case stud*).ti,ab. |
| 37 | conference abstract.pt. |
| 38 | or/34-37 |
| 39 | 33 not 38 |
| 40 | limit 39 to english language |
| 41 | limit 40 to yr="2018 -Current" |

EBM Reviews - Cochrane Database of Systematic Reviews <2005 to November 15, 2023>. Run November 23, 2023

| **Step** | **Search String** |
| --- | --- |
| 1 | [exp cachexia/] |
| 2 | (cachexia or cachectic).mp. |
| 3 | (emaciation or wasting or weight loss or weight reduction).ti,ab. |
| 4 | 1 or 2 or 3 |
| 5 | (neoplasm$ or glioblastoma$ or cancer* or tumour$ or tumor$ or malignan$ or carcinoma$ or adenocarcinoma$ or oncolog$ or gliom$).ti,ab. |
| 6 | 4 and 5 |
| 7 | [exp Activities of Daily Living/] |
| 8 | (daily life activit* or ADL or ADL disabilit*).mp. |
| 9 | [functional status/ or functional assessment.mp.] |
| 10 | [Barthel index/] |
| 11 | [physical performance.mp. or Physical Functional Performance/] |
| 12 | (physical mobilit* or mobility).mp. |
| 13 | ["activities of daily living".mp. or daily life activity/] |
| 14 | "functional independence measure".mp. |
| 15 | [sickness impact profile/] |
| 16 | (ADL* or BADL* or IADL* or EADL*).ti,ab,kw. |
| 17 | (function* adj3 (capacity or independen* or status or disabilit* or assess* or abilit*)).ti,ab,kw. |
| 18 | patient-specific functional scale.ti,ab,kw. |
| 19 | ((daily life or daily living or instrument* or leisure or extended) adj3 activit*).ti,ab,kw. |
| 20 | [exercise test/ or (6MWT or 6MWD or 6 min* walk* test or 6 min* walk* distance or six min* walk* test or six min* walk* distance or walk* test*).ti,ab,kw.] |
| 21 | (Gait speed or walk speed or "timed get up and go" or chair ris* or hand strength or handgrip strength or grip strength).ti,ab,kw. |
| 22 | ((exercise* or physical) and (capacit* or performance* or endurance* or exertion*)).ti,ab,kw. |
| 23 | (sf36 or sf 36 or short form 36 or shortform 36 or shortform36).ti,ab. |
| 24 | (euroqol* or eq5d* or eq 5d*).ti,ab. |
| 25 | (sf12 or sf 12 or short form 12 or shortform 12 or shortform12).ti,ab. |
| 26 | (sf20 or sf 20 or short form 20 or shortform 20 or shortform20).ti,ab. |
| 27 | (sf8 or sf 8 or short form 8 or shortform 8 or shortform8).ti,ab. |
| 28 | [exp "Quality of Life"/] |
| 29 | (quality of life or QoL or QL or HRQoL or HRQL or life quality or health status or health state or health-related or health related or wellbeing or well-being or humanistic).ti,ab,kw. |
| 30 | [exp Patient Reported Outcome Measures/] |
| 31 | ((patient* or proxy or proxies or caregiver*) adj3 (report* or relate* or perspective* or experience* or satisfaction or burden)).ti,ab,kw. |
| 32 | or/7-31 |
| 33 | 6 and 32 |
| 34 | (infan* or child* or adolescen* or pediatr* or paediatr* or juvenile or pregnan*).ti,ab,kw. |
| 35 | [(exp animal/ not (exp human/ and exp animal/)) or Drug Evaluation, Preclinical/] |
| 36 | [(editorial or comment* or letter or note or case study or case studies or case report).pt. or (editorial/ or letter/ or case study/ or case report/ or note/) or (case report* or case stud*).ti,ab.] |
| 37 | conference abstract.pt. |
| 38 | or/34-37 |
| 39 | 33 not 38 |
| 40 | limit 39 to english language [Limit not valid in CDSR; records were retained] |
| 41 | limit 40 to yr="2018 -Current" |

**Supporting Information Tables S2a-S2c**

**Supporting Information Table S2a.** Newcastle-Ottawa Scale for cohort studies (n=20).

| **Author and year** | **Selection** | | | | **Comparability** | **Outcome** | | | **Total** |
| --- | --- | --- | --- | --- | --- | --- | --- | --- | --- |
|  | Representative-ness of the exposed cohort | Selection of the non-exposed cohort | Ascertain- ment of exposure | Demonstration that outcome of interest was not present at start of study | Comparability of cohorts on the basis of the design or analysis | Assessment of outcome | Was follow-up long enough for outcomes to occur? | Adequacy of follow-up of cohorts |  |
|  | **1 pt max** | **1 pt max** | **1 pt max** | **1 pt max** | **2 pts max** | **1 pt max** | **1 pt max** | **1 pt max** |  |
| Cavka 2022 [29] | 0 | 1 | 1 | 1 | 1 | 1 | 1 | 1 | 7 |
| Cavka 2023 [30] | 0 | 1 | 1 | 1 | 1 | 1 | 1 | 1 | 7 |
| Dolan 2020 [52] | 1 | 1 | 1 | 1 | 1 | 1 | 1 | 1 | 8 |
| Evertz 2023 [55] | 0 | 1 | 0 | 1 | 2 | 1 | 1 | 1 | 7 |
| Hadzibegovic 2023 [56] | 0 | 1 | 1 | 1 | 1 | 1 | 1 | 1 | 7 |
| Landgrebe 2023 [58] | 0 | 1 | 1 | 1 | 1 | 1 | 1 | 1 | 7 |
| Matsuo 2023 [59] | 0 | 1 | 1 | 1 | 1 | 1 | 1 | 1 | 7 |
| Miao 2023 [60] | 1 | 1 | 1 | 1 | 1 | 1 | 1 | 1 | 8 |
| Nucci 2023 [61] | 0 | 1 | 1 | 1 | 1 | 1 | 1 | 1 | 7 |
| Roeland 2021 [64] | 0 | 1 | 1 | 1 | 1 | 1 | 1 | 1 | 7 |
| Roy 2023 [65] | 0 | 1 | 1 | 1 | 1 | 1 | 1 | 1 | 7 |
| Song 2021 [66] | 1 | 1 | 1 | 1 | 1 | 1 | 1 | 1 | 8 |
| Ueshima 2023 [40] | 0 | 1 | 1 | 1 | 1 | 1 | 1 | 1 | 7 |
| van der Laan 2021 [70] | 1 | 1 | 1 | 1 | 1 | 1 | 1 | 1 | 8 |
| Wang 2023 [41] | 1 | 1 | 1 | 1 | 0 | 1 | 1 | 1 | 7 |
| Wiegert 2021 [42] | 1 | 1 | 1 | 1 | 2 | 1 | 1 | 1 | 9 |
| Xie 2023 [71] | 1 | 1 | 1 | 1 | 2 | 1 | 1 | 1 | 9 |
| Yi 2023 [72] | 0 | 1 | 1 | 1 | 0 | 1 | 1 | 1 | 6 |
| Zhang 2023 [43] | 1 | 1 | 1 | 1 | 2 | 1 | 1 | 1 | 9 |
| Zhou 2018 [39] | 0 | 1 | 1 | 1 | 0 | 1 | 1 | 1 | 6 |

**Supporting Information Table S2b.** Newcastle-Ottawa Scale for cross-sectional studies (n=18).

| **Author and year** | **Selection** | | | | **Comparability** | **Outcome** | | **Total** |
| --- | --- | --- | --- | --- | --- | --- | --- | --- |
|  | Representative- ness of the sample  (1 for truly or somewhat representative) | Sample size  (1 if sample size calculated and sufficient size; else 0) | Nonrespondents/ response rate  (0 if unsatisfactory recruitment rate or no data on nonrespondents) | Exposure  (1 if obtained from secure record or structured interview; 0 if written self-report or no description) | Comparability of cohorts based on design  (1 for controlling for most important factor, 2 for controlling for 1st and 2nd important factor, 0 for no control) | Outcome assessment  (2 for independent blind assessment or record linkage [claims, charts, EMR], 1 for self-report, 0 for not described) | Statistical test  (1 if used to analyze data with CI or p value; 0 if inappropriate, not described, or none) |  |
|  | **1 pt max** | **1 pt max** | **1 pt max** | **1 pt max** | **2 pts max** | **2 pts max** | **1 pt max** |  |
| Akezaki 2020 [44] | 0 | 1 | 1 | 1 | 0 | 2 | 1 | 6 |
| Alvaro Sanz 2021 [45] | 0 | 1 | 1 | 1 | 1 | 2 | 1 | 7 |
| Amano 2019 [46] | 0 | 1 | 1 | 1 | 0 | 1 | 1 | 5 |
| An 2023 [47] | 0 | 1 | 1 | 1 | 0 | 2 | 1 | 6 |
| Anderson 2020 [27] | 0 | 1 | 1 | 1 | 0 | 2 | 1 | 6 |
| Anderson 2021 [28] | 0 | 1 | 1 | 1 | 0 | 2 | 1 | 6 |
| Antoun 2019 [48] | 1 | 1 | 1 | 1 | 0 | 2 | 1 | 7 |
| Arrieta 2018 [49] | 0 | 1 | 1 | 1 | 1 | 2 | 1 | 7 |
| Cong 2022 [50] | 1 | 1 | 1 | 1 | 2 | 1 | 1 | 8 |
| Daly 2020a [31] | 1 | 1 | 1 | 1 | 1 | 1 | 1 | 7 |
| Daly 2020b [32] | 1 | 1 | 1 | 1 | 1 | 1 | 1 | 7 |
| Demirag 2021 [51] | 1 | 1 | 1 | 0 | 0 | 1 | 1 | 5 |
| Dolin 2023 [53] | 1 | 1 | 1 | 1 | 1 | 1 | 1 | 7 |
| Dunne 2019 [54] | 0 | 1 | 0 | 1 | 2 | 1 | 1 | 6 |
| Kasvis 2019 [57] | 1 | 1 | 1 | 1 | 2 | 1 | 1 | 8 |
| Ohmae 2023 [62] | 0 | 1 | 0 | 1 | 1 | 1 | 1 | 5 |
| Sun 2023 [68] | 1 | 1 | 1 | 1 | 1 | 1 | 1 | 7 |
| Sutandyo 2023 [69] | 0 | 1 | 1 | 1 | 1 | 1 | 1 | 6 |

**Supporting Information Table S2c.** Cochrane Risk of Bias 2 for randomized clinical trials (n=2).

| **Author and year** | **Study design** | **Description / judgement** | **Was the allocation sequence random?** | **Was the allocation sequence concealed until participants were enrolled and assigned to interventions?** | **Was knowledge of the allocated interventions adequately prevented from participants and personnel?** | **Was knowledge of the allocated interventions adequately prevented from outcome assessors?** | **Were incomplete outcome data adequately addressed?** | **Are reports of the study free of suggestion of selective outcome reporting?** | **Was the study apparently free of other problems that could put it at a high risk of bias?** |
| --- | --- | --- | --- | --- | --- | --- | --- | --- | --- |
| Regueme 2021 [63] | RCT  (post hoc) | Description | RCT | NR | Not blinded | Not blinded | Some incomplete data | No evidence of selective reporting | No evidence of other biases |
| Regueme 2021 [63] | RCT  (post hoc) | Judgement | Yes | Unclear | No | No | Unclear | Yes | Yes |
| Stene 2019 [67] | RCT | Description | RCT | NR | Not blinded | Not blinded | No incomplete data | No evidence of selective reporting | No evidence of other biases |
| Stene 2019 [67] | RCT | Judgement | Yes | Unclear | No | No | Yes | Yes | Yes |

Abbreviations: CI, confidence interval; EMR, electronic medical records; max, maximum; NR, not reported; pt, point; RCT, randomized controlled trial.

**Supporting Information Table S3.** Summary of outcomes assessed and statistical significance across all included publications (n=40).

| **Author and year**  **(country); sample size** | **Cachexia or WL definition used** | **Performance status** | **Physical function** | **Activities of daily living** | **HRQoL** | **Depression and/or anxiety** | **Pain** | **Fatigue** |
| --- | --- | --- | --- | --- | --- | --- | --- | --- |
| **Number of publications reporting outcome type, n (%)** | | **16**  **(40%)** | **31**  **(77.5%)** | **6**  **(15%)** | **24**  **(60%)** | **8**  **(20%)** | **14**  **(35%)** | **14**  **(35%)** |
| Akezaki 2020 [44]  (Japan); N=96 | mGPS (Koike 2008) |  |  | **X** |  |  |  |  |
| Alvaro Sanz 2021 [45]  (Spain); N=177 | Fearon et al. 2011 |  | **X** |  | **X** |  |  |  |
| Amano 2019 [46]  (Japan): N=140 | Fearon et al. 2011 |  | O | **X** | **X** | O | O | **X** |
| An 2023 [47]  (China); N=1075 | NR (WL) – no time period, no % reported |  |  |  | **X** |  |  |  |
| Anderson 2020 [27]  (USA): N=75 | Fearon et al. 2011 |  | **X** |  |  |  |  |  |
| Anderson 2021 [28]  (USA): N=133 | Fearon et al. 2011 | **X** | **X** |  | **X** | O | O | O |
| Antoun 2019 [48]  (France and Belgium); N=531 | Fearon et al. 2011 | **X** | **X** |  | **X** |  |  |  |
| Arrieta 2018 [49]  (Mexico); N=200 | NR (WL >10%) |  | **X** |  | **X** |  |  |  |
| Cavka 2022 [29]  (Slovenia); N=141 | Evans et al. 2008 |  |  |  | **X** |  |  |  |
| Cavka 2023 [30]  (Slovenia); N=141 | Evans et al. 2008 |  |  |  | **X** |  |  |  |
| Cong 2022 [50]  (China); N=4231 | Evans et al. 2008 | **X** | **X** |  |  |  |  |  |
| Daly 2020a [31]  (UK and Ireland); N=1027 | Martin et al. 2015 (WLGS) |  | **X** |  | **X** |  | **X** | **X** |
| Daly 2020b [32]  (UK and Ireland); N=1027 | McMillan et al. 2008 (mGPS) and % WL (past 3 mo) |  | **X** |  | **X** |  | **X** | **X** |
| Demirag 2021 [51]  (Turkey); N=174 | Fearon et al. 2011 |  | **X** | **X** | **X** |  | **X** |  |
| Dolan 2020 [52]  (UK and Ireland); N=523 | McMillan et al. 2008 (mGPS) and Martin et al. 2015 (WLGS) |  | **X** |  |  |  |  |  |
| Dolin 2023 [53]  (Denmark); N=64 | Fearon et al. 2011 |  | O |  |  |  |  |  |
| Dunne 2019 [54]  (USA); N=100 | Fearon et al. 2011 |  | O | **X** |  | O |  | O |
| Evertz 2023 [55]  (Germany); N=253 | Fearon et al. 2011^a^ |  | O |  |  |  |  |  |
| Hadzibegovic 2023 [56]  (Germany); N=333 | Fearon et al. 2011 | **X** | **X** |  | **X** |  | **X** |  |
| Kasvis 2019 [57]  (Canada); N=512 | Vigano et al. 2017 |  |  |  | **X** |  |  |  |
| Landgrebe 2023 [58]  (Denmark); N=120 | GLIM criteria: non-volitional WL, low BMI, reduced muscle mass, reduced food intake, inflammation | O | **X** |  | **X** |  | **X** | **X** |
| Matsuo 2023 [59]  (Japan); N=183 | Fearon et al. 2011 | **X** |  |  |  |  |  |  |
| Miao 2023 [60]  (China and Singapore); N=171 | NR (WL ≥ 5%) over 7 wk of Tx |  | **X** |  | **X** |  | O | O |
| Nucci 2023 [61]  (Italy); N=90 | Fearon et al. 2011 |  |  |  |  | **X** |  |  |
| Ohmae 2023 [62-64]  (Japan); N=64 | Fearon et al. 2011 |  | **X** |  |  |  |  |  |
| Regueme 2021 [63]  (France); N=155 | NR (WL), 3 categories (<5%, 5% to <10%, ≥10%) |  | **X** | **X** |  | **X** |  | **X** |
| Roeland 2021 [64]  (USA); N=38 | NR (WL), “weight changes” |  | O |  | O |  |  | O |
| Roy 2023 [65]  (USA); N=250 | Fearon et al. 2011 and Martin et al. 2015 (WLGS) |  |  | **X** |  |  |  |  |
| Song 2021 [66]  (China); N=8466 | Fearon et al. 2011 | **X** | **X** |  |  |  |  |  |
| Stene 2019 [67]  (Norway and UK); N=46 | Fearon et al. 2011 | O | **X** |  |  |  |  |  |
| Sun 2023 [68]  (China); N=528 | Fearon et al. 2011 | **X** | **X** |  | **X** | **X** |  |  |
| Sutandyo 2023 [69]  (Indonesia); N=150 | Fearon et al. 2011 |  | O |  |  |  |  |  |
| Ueshima 2023 [40]  (Japan); N=196 | Zhou et al. 2018 (CSS) | **X** | **X** |  |  |  |  |  |
| van der Laan 2021 [70]  (Netherlands); N=1083 | NR (WL ≥10%) |  |  |  | O |  |  |  |
| Wang 2023 [41]  (China); N=1566 | Wang et al. 2023 (Cancer Cachexia Staging Index, CCSI) | **X** | **X** |  | **X** | **X** | **X** | **X** |
| Wiegert 2021 [42]  (Brazil); N=443 | Wiegert et al. 2021 (Cancer Cachexia Staging System) | **X** | **X** |  | **X** | O | **X** | **X** |
| Xie 2023 [71]  (China); N=16,842 | Modified WLGS (modified from Martin et al. 2015) | **X** | **X** |  | **X** |  | **X** | **X** |
| Yi 2023 [72]  (China); N=270 | Zhou et al. 2018 (CSS, modified to add 3 items [age, BMI, decreased food intake]) | **X** | **X** |  | **X** | **X** | **X** | **X** |
| Zhang 2023 [43]  (China); N=11,423 | Zhang et al. 2023 (Weight Loss and Inflammation Grading System, WLAIGS) | **X** | **X** |  | **X** |  | **X** | **X** |
| Zhou 2018 [39]  (China); N=259 | Zhou et al. 2018 (CSS) | **X** | **X** |  | **X** |  | **X** | **X** |

**X**/O denote that outcomes were assessed; “**X**” indicates a statistically significant association with cachexia or WL for at least 1 measure of the indicated outcome type (a study may have analyzed the same outcome type in different ways or across different patient groups), and “O” indicates that no statistical significance was found.

Abbreviations: BMI, body mass index; CSS, Cachexia Staging Score; GLIM, Global Leadership Initiative on Malnutrition; HRQoL, health-related quality of life; mGPS, modified Glasgow Prognostic Score; NR, not reported; Tx, treatment; WL, weight loss; WLGS, Weight Loss Grading System.

^a^ Study used criteria corresponding to Fearon et al. 2011 but did not identify the criteria as based on Fearon.
